# The Cullin 4A/B-DDB1-Cereblon E3 Ubiquitin Ligase Complex Mediates the Degradation of CLC-1 Chloride Channels

**DOI:** 10.1038/srep10667

**Published:** 2015-05-29

**Authors:** Yi-An Chen, Yi-Jheng Peng, Meng-Chun Hu, Jing-Jia Huang, Yun-Chia Chien, June-Tai Wu, Tsung-Yu Chen, Chih-Yung Tang

**Affiliations:** 1 Department of Physiology, College of Medicine, National Taiwan University, Taipei, Taiwan; 2 Institute of Molecular Medicine, College of Medicine, National Taiwan University, Taipei, Taiwan; 3 Neuroscience Center, University of California, Davis, USA; 4 Graduate Institute of Brain and Mind Sciences, College of Medicine, National Taiwan University, Taipei, Taiwan

## Abstract

Voltage-gated CLC-1 chloride channels play a critical role in controlling the membrane excitability of skeletal muscles. Mutations in human CLC-1 channels have been linked to the hereditary muscle disorder myotonia congenita. We have previously demonstrated that disease-associated CLC-1 A531V mutant protein may fail to pass the endoplasmic reticulum quality control system and display enhanced protein degradation as well as defective membrane trafficking. Currently the molecular basis of protein degradation for CLC-1 channels is virtually unknown. Here we aim to identify the E3 ubiquitin ligase of CLC-1 channels. The protein abundance of CLC-1 was notably enhanced in the presence of MLN4924, a specific inhibitor of cullin-RING E3 ligases. Subsequent investigation with dominant-negative constructs against specific subtypes of cullin-RING E3 ligases suggested that CLC-1 seemed to serve as the substrate for cullin 4A (CUL4A) and 4B (CUL4B). Biochemical examinations further indicated that CUL4A/B, damage-specific DNA binding protein 1 (DDB1), and cereblon (CRBN) appeared to co-exist in the same protein complex with CLC-1. Moreover, suppression of CUL4A/B E3 ligase activity significantly enhanced the functional expression of the A531V mutant. Our data are consistent with the idea that the CUL4A/B-DDB1-CRBN complex catalyses the polyubiquitination and thus controls the degradation of CLC-1 channels.

In skeletal muscles, the voltage-gated CLC-1 chloride (Cl^−^) channel may contribute up to 70%-80% of the resting membrane conductance and thus plays a critical role in controlling the membrane excitability^1^,^2^,^3^. Human CLC-1 is encoded by the *CLCN1* gene on chromosome 7^4^, and more than 100 different mutations in the *CLCN1* gene have been associated with the hereditary muscle disorder myotonia congenita, which is characterized by muscle stiffness after voluntary contraction^5^,^6^,^7^.

The pathophysiological mechanisms of a significant number of myotonia-related mutations can be attributed to the disruption of the gating functions of CLC-1^8^,^9^,^10^,^11^. Several disease-associated *CLCN1* mutations, however, have been shown to yield functional CLC-1 channels with gating properties either only slightly different or virtually indistinguishable from those of wild-type (WT) channels^6^,^12^. In fact, emerging evidence suggests that the effects of myotonia-related CLC-1 mutations may also entail mechanisms other than defective channel gating. For example, a reduced surface expression of CLC-1 channels may underlie the pathology of some myotonia mutations^13^. Moreover, previous studies from other as well as our labs have demonstrated that myotonia-related A531V mutant channel has gating properties similar to those of the WT CLC-1 channel but yields dramatically diminished whole-cell currents due to significantly enhanced protein degradation^14^,^15^,^16^.

It remains unclear with regard to how disease-associated mutant CLC-1 proteins may fail to pass the endoplasmic reticulum (ER) quality control system and thereby display enhanced protein degradation as well as defective membrane trafficking. In addition, the molecular basis of ER-associated degradation (ERAD) for CLC-1 protein is virtually unknown. In ERAD, after being recognized by molecular chaperones, misfolded proteins are subject to ubiquitination by the concerted activity of the ubiquitination machinery, which includes the ubiquitin activating enzyme (E1), the ubiquitin conjugating enzyme (E2), and the ubiquitin ligase (E3)^17^,^18^,^19^,^20^,^21^. To date, over 600 distinct E3 ligases are estimated to express in human cells^22^,^23^. Therefore, one important step to addressing the molecular pathophysiology of myotonia congenita is to elucidate the protein ubiquitination mechanism of CLC-1 channels. In this study, we aim to identify the E3 ubiquitin ligase of CLC-1 channels. Pharmacological and biochemical analyses revealed that two cullin-really interesting new gene (RING) E3 ligases, cullin 4A (CUL4A) and cullin 4B (CUL4B), regulated the protein level and polyubiquitination of CLC-1. In addition, we provided evidence showing that CUL4A/B also modulated the functional expression of CLC-1 channels. Based on our data, we propose that the CUL4A/B E3 ubiquitin ligase mediates the protein degradation of CLC-1 channels.

## Results

### CLC-1 is a substrate protein for CUL4A and CUL4B E3 ubiquitin ligases

**Figure 1:**
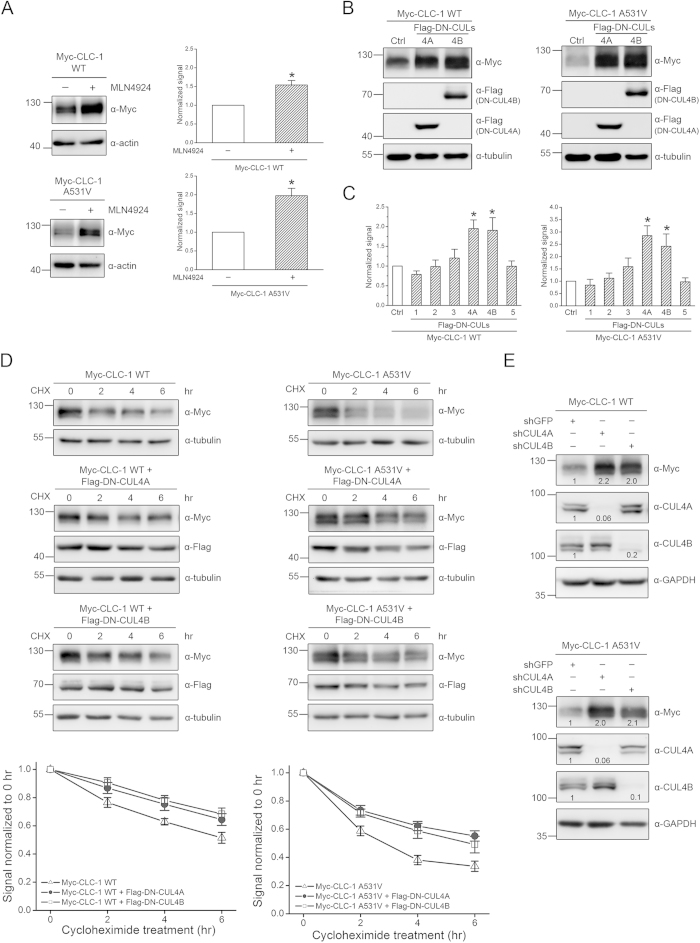
CUL4A and 4B control the protein level of CLC-1. Biochemical demonstration of the regulation of CLC-1 by cullin in HEK293T cells.***(A)**(Left)* Representative immunoblots showing the effect of 24-hr treatment of 10 μM MLN4924 (in 0.1% DMSO) on protein expression of Myc-CLC-1. The molecular weight markers (in kilodaltons) and immunoblotting antibodies (α-Myc and α-actin) are labeled to the left and right, respectively. Expressions of actin are displayed as the loading control. *(Right)* Quantification of relative CLC-1 protein expression level. Protein density was standardized as the ratio of the CLC-1 signal to the cognate actin signal. Values from the MLN4924-treated group (*hatched bars*) were then normalized to those for the corresponding control (*clear bars*). Asterisks denote significant difference from the control (*, *t*-test: p < 0.05; n = 5-6).***(B)*** The effect of Flag-DN-CUL4A/B co-expression on CLC-1. Co-expression with the Flag vector was used as the control experiment (Ctrl). Tubulin was used as the loading control. ***(C)*** Quantification of relative CLC-1 protein level in the presence of various DN-CUL constructs. See Supplementary Figure S1 for more immunoblots. Asterisks denote significant difference from the Flag vector control (Ctrl) (*, *t*-test: p < 0.05; n =4-16). ***(D)**(Top)* The kinetics of CLC-1 protein turn-over in the presence of different treatment durations of 100 μg/ml cycloheximide (CHX). Co-expression with the pcDNA3 vector was used as the control experiment. *(Bottom)* Protein densities were standardized as the ratio of CLC-1 signals to the cognate actin signals, followed by normalization to those of the vector control at 0 hr. Based on linear-regression analyses of the semi-logarithmic plot of the protein degradation time course, the protein half-life values for CLC-1 WT (n = 7-20) are about 6.1 (Control), 9.5 (DN-CUL4A) and 10.8 (DN-CUL4B) hrs; those for the A531V mutant (n = 8-15) are about 3.3 (Control), 5.6 (DN-CUL4A) and 6.5 (DN-CUL4B) hrs.***(E)*** The effect of shRNA knock-down of endogenous CUL4A/B. The numbers denote the relative CLC-1/CUL4A/CUL4B expression level with respect to the control shRNA. The gels were run under the same experimental conditions. Uncropped images of immunoblots are shown in Supplementary Figure S2.

The largest subfamily of human E3 ubiquitin ligases is the cullin RING ligase, estimated to comprise over 200 distinct members^24^,^25^,^26^. To test the hypothesis that cullin RING ligases may regulate CLC-1 biosynthesis, we employed a specific inhibitor of cullin RING E3 ligases, MLN4924^27^,^28^. As illustrated in Fig. 1A, in HEK293T cells transiently over-expressing CLC-1 WT or the myotonia-related A531V mutant, 24-hour treatment with 10 μM MLN4924 induced a significant increase in protein signal, suggesting that cullin-RING ligases may regulate CLC-1 protein expression.

Cullin RING E3 ligases are multi-subunit protein complexes, with the scaffold protein cullin serving as a hub that brings together the catalytic and the substrate-recognition components^24^,^25^,^26^. Human cells express several different subtypes of cullins that each serves as the core of a subclass of cullin-RING ligases. Therefore we tried to determine the specific cullin subtype that contributed to the regulation of CLC-1 protein level by studying the effects of various dominant-negative cullin constructs, which are cullin carboxyl-terminal truncation mutants that retain the substrate recognition capacity but lack the ubiquitin ligase function^29^,^30^,^31^,^32^. Figure 1B,C show that co-expression with dominant-negative cullin 4A (DN-CUL4A) and 4B (DN-CUL4B) constructs resulted in enhanced CLC-1 protein abundance (see also Suppl. Fig. S1). Furthermore, we evaluated the effect of DN-CUL4A/B co-expression on CLC-1 protein stability by using the cycloheximide chase assay. Compared to its WT counterpart, the myotonia-related A531V mutant exhibited significantly enhanced protein degradation^16^. In the presence of DN-CUL4A or DN-CUL4B, however, the protein half-life of A531V was dramatically increased from about 3.3 to about 6.5 hours (Fig. 1D). Likewise, DN-CUL4A/B co-expression considerably augmented the protein half-life of CLC-1 WT, from about 6.1 to about 10.8 hours (Fig. 1D). In addition, shRNA knock-down of endogenous CUL4A/B also enhanced CLC-1 protein expression in HEK293T cells (Fig. 1E).

**Figure 2:**
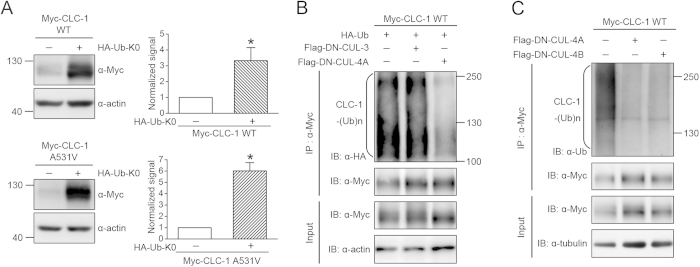
CUL4A and 4B regulate CLC-1 protein polyubiquitination. ***(A)*** Biochemical demonstration of CLC-1 polyubiquitination in HEK293T cells. *(Left)* Representative immunoblots showing the effect of HA-tagged lysine-less ubiquitin (HA-Ub-K0) co-expression on Myc-CLC-1. *(Right)* Quantification of relative CLC-1 protein expression level. Standardized protein densities of the Ub-K0 co-expression group (*hatched bars*) were normalized to those for the corresponding HA-vector control (*clear bars*). Asterisks denote significant difference from the control (*, *t*-test: p < 0.05; n = 5-6).***(B)*** CLC-1 polyubiquitination [CLC-1-(Ub)n] by HA-Ub was reduced by DN-CUL4A, but not DN-CUL3. Co-expression with the Flag vector was used as the control experiment. Cell lysates were immunoprecipitated (IP) with the anti-Myc antibody, and protein ubiquitination was recognized by immunoblotting (IB) the immunoprecipitates with the anti-HA antibody. Corresponding expression levels of CLC-1 and actin in the lysates are shown in the *Input* lane. In all cases hereafter, input represents about 10% of the total protein used for immunoprecipitation. ***(C)*** CLC-1 polyubiquitination by endogenous ubiquitin was disrupted in the presence of DN-CUL4A/B. Co-expression with the Flag vector was used as the control experiment. Protein ubiquitination was identified by immunoblotting the immunoprecipitates with the anti-ubiquitin (Ub) antibody. The gels were run under the same experimental conditions. Uncropped images of immunoblots are shown in Supplementary Figure S2.

In the next line of experiments, we asked whether CUL4A and CUL4B regulated protein ubiquitination of CLC-1 channels. In general, protein ubiquitination involves covalent linkage of lysine residues with either single/multiple monoubiquitins or polyubiquitin chains^33^,^34^. To understand the nature of ubiquitin linkage of CLC-1 channels, we attempted to inhibit polyubiquitin chain elongation in HEK293T cells by over-expressing a lysine-less ubiquitin mutant (Ub-K0) in which all of the lysines were mutated to arginines, thereby averting polyubiquitination of a subset of proteins and consequently preventing their recognition by proteasomes^35^,^36^. Figure 2A shows that co-expression with Ub-K0 resulted in a significant increase in the steady-state level of CLC-1, suggesting that CLC-1 channels are subject to polyubiquitination in HEK293T cells. CLC-1 polyubiquitination was further confirmed by co-expressing CLC-1 with HA-tagged ubiquitin (HA-Ub), wherein immunoblot analyses with the anti-HA antibody revealed a smear of high-molecular weight CLC-1 protein bands (Fig. 2B). Importantly, co-expression with DN-CUL4A, but not DN-CUL3, dramatically attenuated the extent of CLC-1 polyubiquitination by HA-Ub (Fig. 2B). Moreover, using a specific antibody against human ubiquitin, we demonstrated that CLC-1 polyubiquitination by endogenous ubiquitins in HEK293T cells was also notably reduced upon co-expression with DN-CUL4A/B (Fig. 2C).

**Figure 3:**
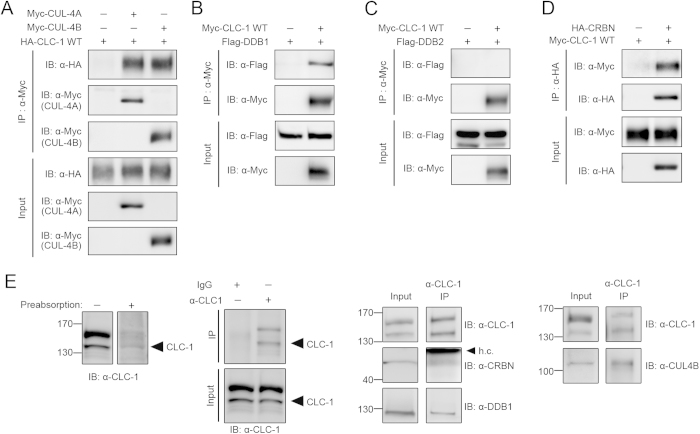
CUL4A/B, DDB1 and CRBN co-exist in the same protein complex with CLC-1. Biochemical inspection of CLC-1 protein interactions. ***(A)*** Co-immunoprecipitation of HA-CLC-1 and Myc-CUL4A/B in HEK293T cells. Co-expression of HA-CLC-1 and the Myc vector was used as the control experiment. ***(B)*** Co-immunoprecipitation of Myc***-***CLC-1 and Flag-DDB1 in HEK293T cells. Co-expression of Flag-DDB1 and the Myc vector was used as the control experiment. ***(C)*** Lack of co-immunoprecipitation of Myc-CLC-1 and Flag-DDB2 in HEK293T cells. Co-expression of Flag-DDB2 and the Myc vector was used as the control experiment. ***(D)*** Co-immunoprecipitation of Myc***-***CLC-1 and HA-CRBN in HEK293T cells. Co-expression of Myc-CLC-1 and the HA vector was used as the control experiment. Cell lysates were immunoprecipitated with the anti-Myc (*A,B,C*) or anti-HA (*D*) antibody. ***(E)*** Protein interactions of endogenous CLC-1 channels in rat skeletal muscle. Verification of the specificity of the anti-CLC-1 antibody: (*Far left*) CLC-1 immunoreactivity was notably reduced in the presence of an excess of a control antigen peptide; (*Center left*) immunoprecipitation was achieved by using the anti-CLC-1 antibody, but not rabbit IgG. (*Center right*, *Far right*) Co-immunoprecipitation of CLC-1, CRBN, DDB1, and CUL4B. Muscle lysates were immunoprecipitated with the anti-CLC-1 antibody. h.c.: IgG heavy chain. The gels were run under the same experimental conditions. Uncropped images of immunoblots are shown in Supplementary Figure S2.

The foregoing observations imply that CUL4A/B catalyses the polyubiquitination and thus controls the protein turnover of CLC-1 channels. To further investigate the potential interaction between CUL4A/B and CLC-1 channels, we co-expressed CLC-1 and CUL4A/B in HEK293T cells. As depicted in Fig. 3A, CUL4A/B was indeed co-immunoprecipitated with CLC-1, consistent with the idea that CUL4A/B co-existed with CLC-1 in the same protein complex.

### CRBN regulates CLC-1 protein stability and ubiquitination

The typical substrate-recognition component of cullin E3 ligases comprises an adaptor protein and a substrate receptor protein^24^,^25^,^26^. The adaptor protein links the cullin protein with the substrate receptor, which in turns determines the substrate specificity of a given cullin E3 ligase complex. Damage-specific DNA binding protein 1 (DDB1) is the canonical adaptor protein of CUL4A/B^37^,^38^. Therefore, if CUL4A/B really catalyses CLC-1 ubiquitination, then DDB1 is likely to form a protein complex with CLC-1 channels. Figure 3B illustrates that DDB1 was indeed co-immunoprecipitated with CLC-1.

So far, our data are consistent with the presence of a protein complex comprising CUL4A/B, DDB1, and CLC-1. We further addressed the molecular nature of this putative complex by searching for the substrate receptor linking CUL4A/B-DDB1 with CLC-1. To date, over 50 proteins have been suggested to serve as potential substrate receptors for CUL4A/B-DDB1^39^,^40^,^41^,^42^. For example, damage-specific DNA binding protein 2 (DDB2) is a well recognized CUL4A/B-DDB1 substrate receptor responsible for the ubiquitination of nucleotide excision repair protein. As illustrated in Fig. 3C, however, DDB2 was not co-immunoprecipitated with CLC-1.

Cereblon (CRBN) was previously identified as a novel CUL4A substrate receptor^43^,^44^. Interestingly, a glutathione S-transferase (GST) pull-down assay revealed that *in vitro* translated CRBN appeared to physically interact with a GST fusion protein containing the carboxyl terminus of CLC-1 protein^45^. To determine whether CRBN and CLC-1 may interact with each other in their native protein conformations, we co-expressed CRBN and CLC-1 in HEK293T cells. Figure 3D clearly demonstrates that CRBN was co-immunoprecipitated with CLC-1. Remarkably, endogenous CUL4B, DDB1, CRBN, and CLC-1 proteins in rat skeletal muscle tissues seemed to co-exist in the same protein complex as well (Fig. 3E). In addition, treatment of skeletal muscle explant with MLN4924 resulted in considerable increase in CLC-1 protein expression (Suppl. Fig. S3).

**Figure 4:**
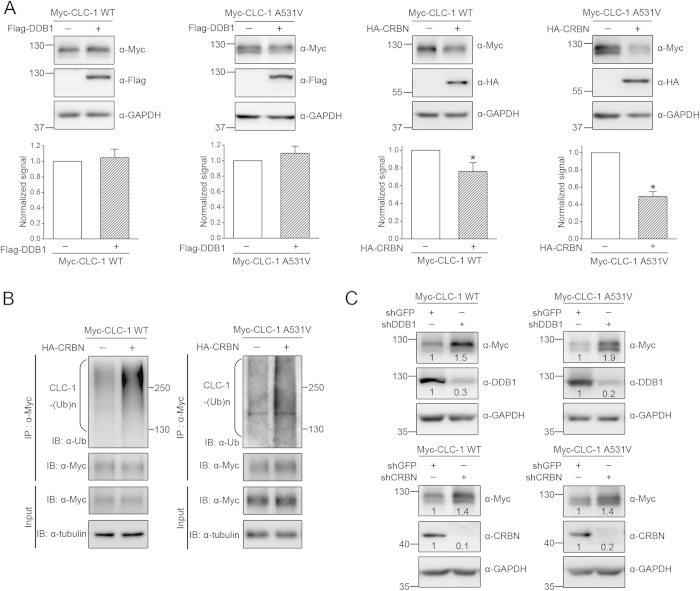
CRBN modulates CLC-1 protein level. Biochemical demonstration of the regulation of CLC-1 by CRBN in HEK293T cells. ***(A)**(Top)* Representative immunoblots showing the effect of Flag-DDB1 or HA-CRBN over-expression on Myc-CLC-1 protein. Co-expression with the Flag/HA vector was used as the control experiment. *(Bottom)* Quantification of relative CLC-1 protein expression level. Values from the DDB1/CRBN co-expression group (*hatched bars*) were normalized to those for the corresponding vector control (*clear bars*). Asterisks denote significant difference from the control (*, *t*-test: p < 0.05; n =6-12).***(B)*** CLC-1 polyubiquitination [CLC-1-(Ub)n] by endogenous ubiquitin was enhanced by CRBN over-expression. Cell lysates were immunoprecipitated (IP) with the anti-Myc antibody. Protein ubiquitination was identified by immunoblotting the immunoprecipitates with the anti-ubiquitin (Ub) antibody. ***(C)*** Representative immunoblots showing the effect of shRNA knock-down of endogenous DDB1 or CRBN. The numbers denote the relative CLC-1/DDB1/CRBN expression level with respect to the control shRNA for GFP. The gels were run under the same experimental conditions. Uncropped images of immunoblots are shown in Supplementary Figure S2.

We then investigated the role of CRBN in the biosynthesis of CLC-1 channels by co-expressing CRBN with CLC-1 in HEK293T cells. Over-expressing CRBN notably reduced the total protein of CLC-1 (Fig. 4A). By contrast, over-expressing DDB1 did not seem to affect CLC-1 protein expression (Fig. 4A), which may imply that HEK293T cells are endowed with a high expression level of endogenous DDB1. Since the protein half-life of CRBN was much shorter than that of CLC-1 (Suppl. Fig. S4), we failed to evaluate the effect of CRBN over-expression on CLC-1 protein turn-over with the cycloheximide chase assay. Nevertheless, CRBN over-expression enhanced the polyubiquitination of CLC-1 channels (Fig. 4B). Moreover, shRNA knock-down of endogenous DDB1 or CRBN resulted in increased CLC-1 protein expression in HEK293T cells (Fig. 4C–D). Together these observations support the notion that CRBN contributes to the regulation of CLC-1 protein stability and ubiquitination.

### Suppression of CUL4A/B E3 ligase activity enhances CLC-1 functional expression

**Figure 5:**
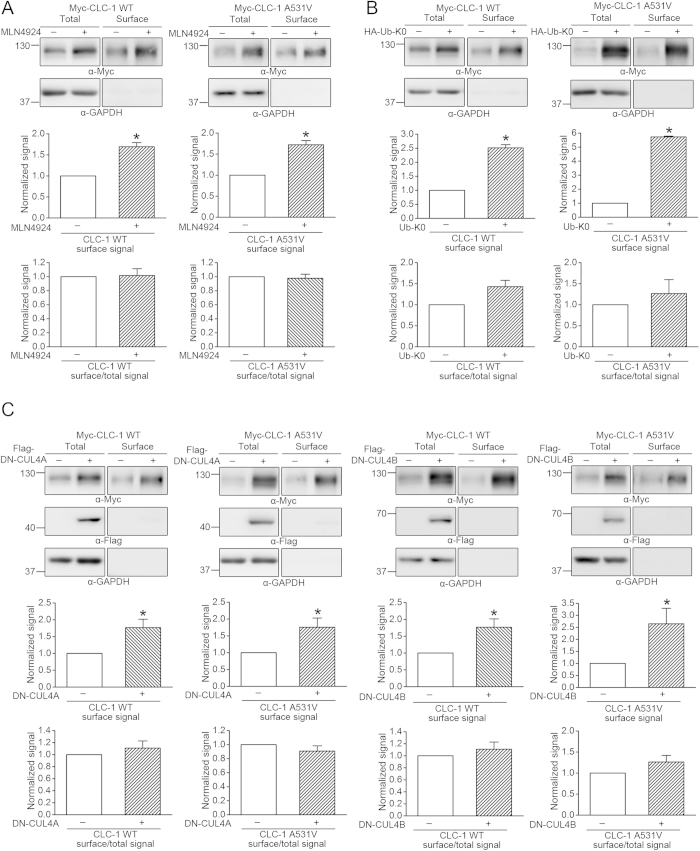
Suppression of CUL4A/B E3 ligase activity increases CLC-1 surface expression. Surface biotinylation experiments on HEK293T cells expressing Myc-CLC-1 channels in the presence of ***(A)*** 10 μM MLN4924, ***(B)*** HA-Ub-K0, or ***(C)*** Flag-DN-CUL4A/B. Drug-free incubation or co***-***expression with the HA/Flag vector was used as the control experiment. (*Top*) Representative immunoblots. Cell lysates from biotinylated intact cells were either directly employed for immunoblotting analyses (total) or subject to streptavidin pull-down before being used for immunoblotting analyses (surface). (*Middle*) Quantification of surface protein level. The surface protein density was standardized as the ratio of surface signal to cognate total actin signal, followed by normalization to that of the corresponding control. Asterisks denote significant difference from the control (*, *t*-test: p < 0.05; n = 3-9). (*Bottom*) Quantification of surface expression efficiency. The total protein density was standardized as the ratio of input signal to actin signal. The efficiency of surface presentation was expressed as surface protein density divided by the corresponding standardized total protein density. The mean surface expression ratio was normalized to that of the corresponding control. The gels were run under the same experimental conditions. Uncropped images of immunoblots are shown in Supplementary Figure S2.

We have previously demonstrated that treatment with the proteasome inhibitor MG132 considerably raised the protein expression of the myotonia-related A531V mutant; the majority of the rescued A531V protein, however, was trapped in the cytoplasm and failed to reach the plasma membrane^16^. By contrast, MG132 treatment enhanced both the protein level and the functional expression of CLC-1 WT. Consequently, a key question that we need to address here is whether inhibition of protein ubiquitination may instead be able to boost the surface expression of the A531V mutant. To investigate the membrane trafficking property of CLC-1 channels, we began by performing protein biotinylation assays. Figure 5A,B show that in the presence of MLN4924 or Ub-K0, CLC-1 WT and A531V displayed comparable increase in cell surface expression; in other words, unlike the effect of MG132 treatment, neither MLN4924 treatment nor Ub-K0 co-expression appreciably disrupted the membrane trafficking efficiency of A531V. Similarly, co-expression with DN-CUL4A/B effectively enhanced the membrane expression of both the WT protein and the A531V mutant (Fig. 5C).

**Figure 6:**
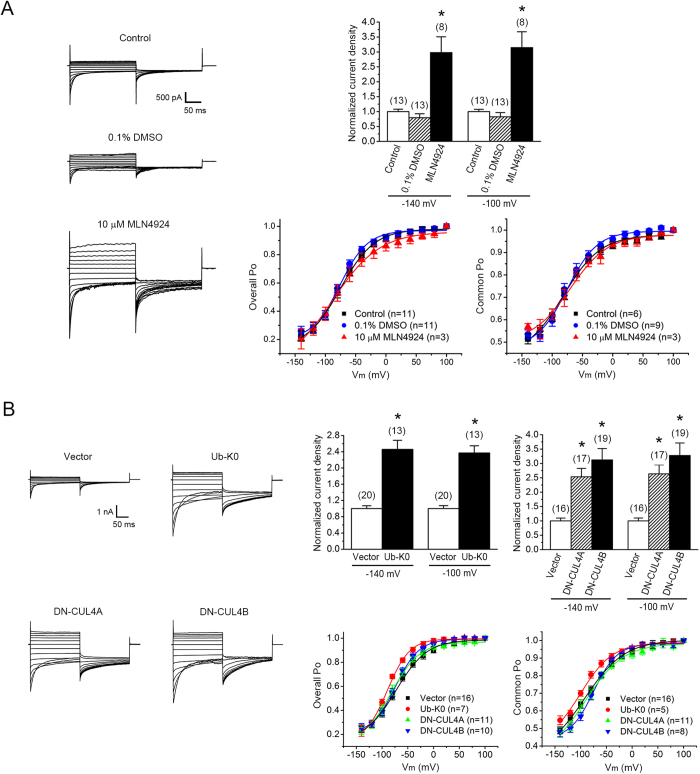
Suppression of CUL4A/B E3 ligase activity enhances the functional expression of the A531V mutant. Electrophysiological analyses of Flag-CLC-1 A531V channels in HEK293T cells.***(A)*** Treatment with 10 μM MLN4924 (in 0.1% DMSO) increased the current amplitude of the A531V mutant. *(Left)* Representative whole-cell patch clamp recordings. Two types of drug-free incubation (control and 0.1% DMSO) were used to evaluate the effect of MLN4924. The holding potential was 0 mV. The voltage protocol comprised a 200-ms test pulse (Vm) ranging from +100 mV to -140 mV in -20 mV steps, followed by a second step (tail potential) to -100 mV for 200 ms. *(Upper right)* Instantaneous current amplitudes at the test pulse potential of -140 or -100 mV were used for the calculation of whole-cell current density, followed by normalization with respect to the corresponding control condition. Despite the presence of some peak current amplitudes over 10 nA, voltage clamping or filtering errors did not appear to notably affect the validity of our analyses (see Supplementary Figure S5). *(Lower right)* Steady-state voltage-dependence properties (*P*_o_–V curves) of the A531V mutant. Both the *P*_o_ of fast and common gates (Overall *P*_o_) and the *P*_o_ of common gates (Common *P*_o_) were analyzed. See Supplementary Table S1 for more details on *P*_o_–V parameters.***(B)*** Co-expression with Ub-K0 or DN-CUL4A/B increased the current amplitude of the A531V mutant. Co-expression with the HA/Flag vector was used as the control experiment. *(Left)* Representative whole-cell patch clamp recordings. *(Upper right)* Normalized instantaneous current densities. *(Lower right)* Steady-state voltage-dependence properties. Asterisks denote a significant difference from the control condition (*, *t*-test: p < 0.05).

The aforementioned observations suggested that the A531V protein spared from polyubiquitination seemed to be properly ushered to the plasma membrane. Nevertheless, can the spared mutant protein form functional Cl^-^ channels in the cell surface? As depicted in Fig.6 and Supplementary Figure S5, the whole-cell Cl^-^ current density of the A531V mutant was notably increased in the presence of MLN4924, Ub-K0, or DN-CUL4A/B, suggesting that the A531V protein spared from polyubiquitination indeed produced functional CLC-1 channels. Similarly, suppression of CUL4A/B E3 ligase activity markedly enhanced the functional expression of CLC-1 WT channels as well (Suppl. Fig. S6).

## Discussion

The A531V mutant is a myotonia congenita-associated human CLC-1 channel mutation found in significant prevalence in northern Finland and northern Scandinavia^14^,^46^. This CLC-1 mutant displays dramatically enhanced proteasomal protein degradation, thereby manifesting a diminished whole-cell current density and a reduction in the total protein level^15^,^16^. These observations are consistent with the idea that A531V is endowed with a folding anomaly that makes the mutant channel undesirable for the protein quality control system in ER, thus leading to a bias of the biosynthetic balance tilted toward the degradation pathway.

To the best of our knowledge, no previous research has ever addressed the molecular machinery responsible for the quality control and protein ubiquitination of CLC-1 channels. As an important step toward understanding why the majority of the newly synthesized A531V protein fail to pass the protein quality control system, in the present study we aim to identify the E3 ubiquitin ligase for CLC-1 channels. Pharmacological and biochemical inhibition of CUL4A/B notably reduced CLC-1 protein turn-over and ubiquitination. Co-immunoprecipitation analyses further revealed that CLC-1 co-existed in the same protein complex with CUL4A/B, as well as the previously identified CUL4A/B-associated binding partners DDB1 and CRBN. Disruption of DDB1 or CRBN expression also resulted in enhanced CLC-1 protein level. Taken together, we propose that the CUL4A/B-DDB1-CRBN E3 ligase complex catalyses the ubiqutination and regulates the degradation of CLC-1 channels.

Cullin RING E3 ligases are multi-subunit protein complexes, consisting of the scaffold protein cullin that binds to a RING protein (which links with an E2 ubiquitin conjugating enzyme to perform the catalytic function), and an adaptor protein (which recruits a substrate receptor to determine the substrate specificity)^24^,^25^,^26^. The human cullin is a family of seven different subtypes (CUL1, CUL2, CUL3, CUL4A, CUL4B, CUL5, and CUL7), each forming the core of a subclass of cullin-RING E3 ligases. To date, about 90 different CUL4A/B E3 ubiquitin ligase complexes have been identified in mammals, about 60 of which incorporate DDB1 as the adaptor protein^37^,^42^. Interestingly, a substrate protein may be subject to modulation by more than one type of E3 ligase; for instance, human ether-à-go-go-related K^+^ channels were suggested to be differentially regulated by the E3 ubiquitin ligases CHIP and Nedd4-2^47^,^48^,^49^,^50^. Moreover, at least seven distinct types of E3 ligases appear to contribute to the ubiquitination of another human Cl^-^ channel, cystic fibrosis transmembrane conductance regulator^51^,^52^. Therefore, we cannot completely rule out the possibility that CLC-1 may also bind to other CUL4A/B-DDB1 substrate receptors, or that CLC-1 ubiquitination may be mediated by E3 ligases other than cullin RING proteins as well.

Via diverse substrate receptors, CUL4A/B-DDB1 E3 ligases have been associated with the regulation of a wide variety of different cellular functions, such as DNA replication and damage recognition, cell cycle control, steroid receptor activation, and tumor suppression. Nevertheless, even for a given substrate receptor, the precise role of CUL4A/B-DDB1 in the regulation of protein ubiquitination appears to be substrate-dependent and may be surprisingly divergent. For example, in addition to DDB1^43^,^44^, CRBN has been suggested to interact with ion channels^45^,^53^, protein kinase^54^, and transcription factors^55^,^56^. CUL4A/B-DDB1 promoted auto-ubiquitination of CRBN, which was suppressed in the presence of the teratogenic drug thalidomide^44^. By contrast, in the presence of lenalidomide, a thalidomide analogue that is used for treating multiple myeloma, CUL4A/B-DDB1-CRBN was induced to acquire the capacity to catalyse the selective ubiquitination and degradation of two lymphoid transcription factors^55^,^56^. On the other hand, despite its potential interaction with CRBN, AMP-activated protein kinase, a multifunctional metabolic sensor essential for cell survival, did not seem to be regulated by CUL4A/B-DDB1^54^.

The large-conductance Ca^2+^-activated K^+^ channel was the first identified binding partner of CRBN 53. Nonetheless, the CUL4A-DDB1-CRBN E3 ligase complex failed to promote the degradation of the K^+^ channel; instead, protein ubiquitination of the K^+^ channel resulted in enhanced ER retention and thus reduced membrane expression^57^. Moreover, by means of yeast two-hybrid screening of a bovine retina cDNA library, CRBN was previously identified as a potential binding partner of CLC-2 protein, another member of the voltage-gated CLC Cl^-^ channel family^45^. Further GST pull-down assays revealed that the carboxyl terminus of both CLC-1 and CLC-2 channels seemed to bind to the Lon domain of CRBN. However, neither biochemical nor functional characterizations were performed to determine whether CRBN controlled the protein expression of CLC-1 or CLC-2 channels. Therefore, to the best of our knowledge, the current study provides the first direct evidence showing that the CUL4A/B E3 ligase complex mediates protein degradation of ion channels.

Numerous disease-associated mutations have been documented to disrupt protein homeostasis^58^,^59^. Prior work from our lab has shown that the major defects of the myotonia-related A531V mutant appear to occur mainly in the biosynthesis of the channel protein^16^. In accordance with this notion, 24-hour treatment with the proteasome inhibitor MG132 dramatically recovered the defective total protein expression of A531V. Nevertheless, MG132 treatment failed to boost the Cl^-^ current density of A531V-transfected cells. Further biochemical and morphological analyses revealed that the majority of the A531V protein spared from proteasomal degradation was still rejected from the membrane trafficking pathway. By contrast, in the current study, we found that the A531V protein spared from polyubiquitination seemed to be properly ushered to the plasma membrane and form functional Cl^-^ channels. Together, these observations raise an intriguing possibility that suppression of the ubiquitination of the A531V mutant may somehow facilitate the protein re-folding process or alter the stringency of the protein quality control system, thereby promoting the surface expression of the mutant channel. MLN4924, which inactivates cullin E3 ligases by blocking the conjugation of the ubiquitin-like molecule Nedd8 to the cullin scaffold protein^27^,^28^, has recently emerged as a novel anti-cancer agent 60,61,62. Our demonstration that suppression of ubiquitination effectively enhanced the protein level as well as the functional expression of the A531V mutant may shed light on the therapeutic potential of cullin inhibitor in correcting disease-related protein expression defects in CLC-1 channels.

Disruption of coordination in the activity of ER folding, quality control, and degradation machineries can result in defective protein maturation, which is linked to the pathogenesis of many human diseases^63^,^64^,^65^. Since myotonia congenita-associated mutations may involve enhanced protein degradation and reduced surface expression, it is of high therapeutic significance to decipher the signaling mechanisms as well as the protein machinery essential for CLC-1 biosynthesis. The discovery of the critical role of the CUL4A/B-DDB1-CRBN E3 ligase complex in the conformation surveillance system of CLC-1 protein may pave the way for future identification of additional components (for example, molecular chaperones) of the molecular machinery mediating the protein homeostasis of CLC-1 in various cellular compartments.

## Methods

### cDNA constructs

Human CLC-1 cDNA in the pcDNA3 vector (Invitrogen) was used to create the myotonia-associated A531V mutant as described previously^16^. Myc- and HA-tagged CLC-1 constructs were generated by inserting the epitope sequence between the residues G438 and D439 in the extracellular linker between L and M helices. For N-terminal Flag-tagged constructs, CLC-1 cDNA was subcloned into the pFlag-CMV2 vector (Sigma).

Other cDNA constructs employed in this study include pcDNA3.1-Flag dominant-negative human cullin 1/2/3/4A/4B/5 (Addgene 15818-15823), pcDNA3-Myc human cullin 3/4A/4B (Addgene 19893, 19951, 19922), pcDNA3-HA wild-type and lysine-less human ubiquitin (kindly provided by Dr. Chihiro Sasakawa, University of Tokyo, Japan), pcDNA3-Flag human DDB1 (Addgene 19918), pcDNA3-Flag human DDB2 (kindly provided by Dr. Show-Li Chen, National Taiwan University, Taiwan), and pcDNA3-HA rat cereblon (kindly provided by Dr. Chul-Seung Park, Gwangju Institute of Science and Technology, Korea).

### Cell culture and DNA transfection

Human embryonic kidney (HEK) 293T cells were grown in Dulbecco’s modified Eagle’s medium (DMEM) supplemented with 2 mM glutamine, 10% heat-inactivated fetal bovine serum (Hyclone), 100 units/ml penicillin, and 50 μg/ml streptomycin, and were maintained at 37 °C in a humidified incubator with 95% air and 5% CO_2_. Transient transfection was performed by using the Lipofectamine 2000 (LF2000) reagent (Invitrogen). Briefly, cells were plated onto 6- or 12-well plates (for biochemical experiments) or poly-D-lysine-coated coverslips in 24-well plates (for electrophysiological recordings) 24 hrs before transfection. Various expression constructs were incubated with LF2000 reagent for 20 min at room temperature, and DNA-lipofectamine diluted in Opti-MEM (Invitrogen) was added to culture wells. After 6-hr incubation at 37 °C, the medium was changed and the culture cells were maintained in the 37 °C incubator for^24^,^25^,^26^,^27^,^28^,^29^,^30^,^31^,^32^,^33^,^34^,^35^,^36^,^37^,^38^,^39^,^40^,^41^,^42^,^43^,^44^,^45^,^46^,^47^,^48^ hrs before being used for biochemical or electrophysiological experiments. Where indicated, drugs [MLN4924 (kindly provided by Dr. Kuo-How Huang, National Taiwan University, Taiwan), MG132 (Sigma), or cycloheximide (Sigma)] were applied to the culture medium.

### RNA interference

Lentivirus-based shRNA constructs (subcloned into the pLKO.1 vector) targeting specific human CUL4A (5’-GCAGAACTGATCGCAAAGCAT-3’), CUL4B (5’-GCCATGAAAGAAGCATTTGAA-3’), DDB1 (5’-GCCTGCATCCTGGAGTATAAA-3’), or CRBN (5’-GCTGCTTGTCTTCCTATTGAT-3’) sequence were purchased from National RNAi Core Facility, Taiwan. The shRNA for GFP (5’-CAACAGCCACAACGTCTATAT-3’) was used as a control. Recombinant lentivirus was generated by co-transfecting HEK293T cells with the packaging plasmid pCMV-ΔR8.91, the envelope plasmid pMD.G, and shRNA expressing constructs. The virus-containing supernatant was harvested and concentrated by ultracentrifugation to yield the viral stock, which in turn was supplemented with 8 μg/ml of polybrene for infection of HEK293T cells. The infected cells were selected by 5 μg/ml of puromycin and subsequently transfected with the cDNA for CLC-1.

### Immunoblotting

Transfected HEK293T cells were washed twice with ice-cold PBS [(in mM) 137 NaCl, 2.7 KCl, 4.3 Na_2_HPO_4_ · 2H_2_O, 1.4 KH_2_PO_4_, pH 7.3] supplemented with 2 mM EDTA, and resuspended in a lysis buffer [(in mM) 150 NaCl, 5 EDTA, 50 Tris-HCl pH7.6, 1% Triton X-100) containing a protease inhibitor cocktail (Roche Applied Science). After adding the Laemmli sample buffer to the lysates, samples were sonicated on ice (three times for five seconds each) and heated at 70 °C for 5 min. Samples were then separated by 7.5-10% SDS-PAGE, electrophoretically transferred to nitrocellulose membranes, and detected using mouse anti-β-actin (1:5000; Millipore), rabbit anti-CLC-1 (1:500; Alomone), rabbit anti-CUL4A (1:2000; GeneTex), rabbit anti-CUL4B (1:1000; ProteinTech), rabbit anti-CRBN (1:1000; Aviva), rabbit anti-DDB1 (1:3000; GeneTex), rabbit anti-Flag (1:5000; Sigma), rabbit anti-GAPDH (1:8000; GeneTex), rabbit anti-HA (1:8000; GeneTex), mouse anti-Myc (clone 9E10), rabbit anti-α-tubulin (1:5000; GeneTex), or mouse anti-ubiquitin (FK2; 1:1000; Enzo Life Sciences) antibodies. Blots were then exposed to horseradish peroxidase-conjugated anti-mouse/rabbit IgG (1:5000; Jackson ImmunoResearch), and revealed by an enhanced chemiluminescence detection system (Thermo Scientific). Results shown are representative of at least three independent experiments. Densitometric scans of immunoblots were quantified by using ImageJ (National Institute of Health).

### Co-immunoprecipitation

Transfected cells were incubated at 37 °C in the presence of 10 μM MG132 for 24 hrs. Cells were solubilized in ice-cold IP buffer [(in mM) 100 NaCl, 4 KCl, 2.5 EDTA, 20 NaHCO_3_, 20 Tris-HCl, pH 7.5, 1 dithiothreitol, 1 phenylmethylsulfonyl fluoride, 1% Triton X-100] containing the protease inhibitor cocktail. Insolubilized materials were removed by centrifugation. Solubilized lysates were pre-cleared with protein G sepharose beads (GE Healthcare Biosciences) for 1 hr at 4 °C, and then incubated for 16 hrs at 4 °C with protein G sepharose beads pre-coated with the anti-Myc or anti-HA antibody. Beads were gently spun down and washed twice in a wash buffer [(in mM) 100 NaCl, 4 KCl, 2.5 EDTA, 20 NaHCO_3_, 20 Tris-HCl, pH 7.5] supplemented with 0.1% Triton X-100, and then twice with the wash buffer. The immune complexes were eluted from the beads by heating at 70 °C for 5 min in the Laemmli sample buffer.

### Preparation of rat skeletal muscle for co-immunoprecipitation

Skeletal muscle fibers were isolated from adult Wistar rats. Animals were anesthetized and sacrificed by procedures that are in accordance with the Guidelines for the Care and Use of Mammals in Neuroscience and Behavioral Research (National Research Council 2003) and approved by the Institutional Animal Care and Use Committee (IACUC) of College of Medicine, National Taiwan University. In brief, vastus lateralis (thigh muscle) was dissected and homogenized in the T-PER tissue extraction reagent (Thermo Scientific; 0.1 g muscle per 500 μl reagent). Lysates were cleared by micro-centrifugation at 13,000 *rpm* for 15 min. The supernatants were pre-cleared with protein A sepharose beads (GE Healthcare Biosciences) for 2 hrs, incubated with the anti-CLC-1 antibody at 4 °C for 2 hrs, and then incubated with protein A sepharose beads at 4 °C for 16 hrs. Beads were gently spun down and washed twice in the T-PER reagent and then three times with the wash buffer. The immune complexes were eluted from the beads by heating at 70 °C for 5 min in the Laemmli sample buffer. Where indicated, the specificity of immunoprecipitation was verified with mouse IgG (Sigma).

### Protein ubiquitination analyses

Transfected cells were incubated at 37 °C in the presence of 10 μM MG132 for 24 hrs. Cells were solubilized in the IP buffer supplemented with 2.5 mg/ml N-Ethylmaleimide, followed by immunoprecipitation with the anti-Myc antibody as described above.

### Biotinylation of cell surface proteins

Transfected cells were washed extensively with D-PBS (Sigma) supplemented with 0.5 mM CaCl_2_, 2 mM MgCl_2_, followed by incubation in 1 mg/ml sulfo-NHS-LC-biotin (Thermo Scientific) in D-PBS at 4 °C for 1 hr with gentle rocking. Biotinylation was terminated by removing the biotin reagents and rinsing three times with 100 mM glycine in PBS, followed by once in TBS buffer [(in mM) 20 Tris-HCl, 150 NaCl, pH 7.4]. Cells were solubilized in a lysis buffer [(in mM) 150 NaCl, 50 Tris-HCl, 1% Triton X-100, 5 EDTA, 1 phenylmethylsulfonyl fluoride, pH 7.6] supplemented with the protease inhibitor cocktail. Insolubilized materials were removed by centrifugation. Solubilized cell lysates were incubated overnight at 4 °C with streptavidin-agarose beads (Thermo Scientific). Beads were washed once in the lysis buffer, followed by twice in a high-salt buffer [(in mM) 500 NaCl, 5 EDTA, 50 Tris-HCl, pH7.6, 0.1% Triton X-100] and once in a low-salt buffer [(in mM) 2 EDTA, 10 Tris-HCl, pH7.6, 0.1% Triton X-100]. The biotin-streptavidin complexes were eluted from the beads by heating at 70 °C for 5 min in the Laemmli sample buffer.

### Electrophysiological recordings

Conventional whole-cell and cell-attached patch clamp techniques were employed to record CLC-1 Cl^−^ currents. Cells co-transfected with the cDNA for pEGFP and Flag-tagged CLC-1 (molar ratio 1:10) were identified with an inverted fluorescence microscope (Leica-DM IRB). Recording electrodes were pulled by a PP-830 puller (Narashige), and displayed a resistance of 2-3 MΩ when filled with the pipette solution. For whole-cell recordings, the pipette solution contained (in mM): 120 CsCl, 10 EGTA, 10 HEPES, pH 7.4; while the bath solution comprised (in mM): 140 NaCl, 4 CsCl, 2 MgCl_2_, 2 CaCl_2_, 10 HEPES, pH 7.4. For cell-attached recordings, the pipette solution was the same as the whole-cell bath solution; whereas the bath solution contained (in mM): 130 KCl, 5 MgCl_2_, 1 EGTA, 10 HEPES, pH 7.4. Data were acquired and digitized with Axopatch 200B and Digidata 1322A, respectively, via pCLAMP 9.0 (Molecular Devices). Cell capacitances were measured using the built-in functions of pCLAMP 9.0 and were compensated electronically with Axopatch 200B. The holding potential was set at 0 mV. Data were sampled at 2 kHz and filtered at 1 kHz. All recordings were performed at room temperature (20-22 °C).

### Statistical analyses

All values were presented as mean ± SEM. The significance of the difference between two means was tested using the Student’s *t* test, whereas means from multiple groups were compared using the one-way ANOVA analysis. All statistical analyses were performed with Origin 7.0 (Microcal Software).

## Additional Information

**How to cite this article**: Chen, Y.-A. *et al.* The Cullin 4A/B-DDB1-Cereblon E3 Ubiquitin Ligase Complex Mediates the Degradation of CLC-1 Chloride Channels. *Sci. Rep.* **5**, 10667; doi: 10.1038/srep10667 (2015).

## Supplementary Material

Supporting Information
