# Supplementary material for: The Cullin 4A/B-DDB1-Cereblon E3 Ubiquitin Ligase Complex Mediates the Degradation of CLC-1 Chloride Channels
Source: Sci Rep. 2015 May 29;5:10667. doi: 10.1038/srep10667 (PMC4448132; doi:10.1038/srep10667)

**Supplementary Information**

**The Cullin 4A/B-DDB1-Cereblon E3 Ubiquitin Ligase Complex Mediates the Degradation of CLC-1 Chloride Channels**

Yi-An Chen, Yi-Jheng Peng, Meng-Chun Hu, Jing-Jia Huang, Yun-Chia Chien, June-Tai Wu, Tsung-Yu Chen, and Chih-Yung Tang

***Supplementary Table S1. Steady-state voltage-dependent activation parameters of CLC-1 channels.*** (related to Figure 6)

***Electrophysiological experiments were conducted to obtain the voltage-dependence of the open probability (P_o_–V curve). To estimate the P_o_ of both fast and common gates (Overall P_o_; P_f_ x P_c_) of CLC-1 channels, the standard voltage protocol (see Fig. 6A) was employed to measure tail current amplitudes at -100 mV. For the P_o_ of common gates (Common P_o_; P_c_), the -100-mV tail potential was preceded by a 1-ms +170-mV pulse to fully activate fast gates. The value of the initial tail current amplitude, determined by fitting the tail current to a double-exponential function, was normalized to the maximal initial tail current amplitude obtained following the +100-mV test pulse. Data points in the P_o_-V curve were fitted with a Boltzmann equation: P_o_ = P_min_ + (1-P_min_) / {1+exp[(V_0.5_-V)/k]}, where V_0.5_ and k is the half-activating voltage and slope factor, respectively, for the P_o_-V curve.***

|  | | Overall Po (P_f_ x P_c_) | | | Common Po (P_c_) | | |
| --- | --- | --- | --- | --- | --- | --- | --- |
|  |  | V_0.5_ (mV) | *k* | n | V_0.5_ (mV) | *k* | n |
| **A531V (whole-cell)** | Control | -75.4 ± 2.5 | 28.1 ± 1.9 | 11 | -81.4 ± 4.2 | 30.8 ± 2.9 | 6 |
|  | 0.1% DMSO | -76.0 ± 2.8 | 22.9 ± 2.2 | 11 | -75.5 ± 4.2 | 25.1 ± 3.3 | 9 |
|  | MLN4924 | -88.1 ± 8.9 | 38.7 ± 5.4 | 3 | -69.9 ± 6.6 | 31.0 ± 5.2 | 3 |
|  | Vector | -72.6 ± 2.8 | 30.0 ± 2.1 | 16 | -92.7 ± 3.1 | 38.9 ± 1.7 | 16 |
|  | Ub-K0 | -89.6 ± 2.1 | 21.7 ± 1.5 | 7 | -101.0 ± 7.7 | 31.6 ± 4.0 | 5 |
|  | DN-CUL4A | -81.9 ± 3.0 | 27.5 ± 2.2 | 11 | -90.5 ± 8.7 | 36.9 ± 5.1 | 11 |
|  | DN-CUL4B | -73.9 ± 1.5 | 22.9 ± 1.2 | 10 | -74.8 ± 2.1 | 23.0 ± 1.7 | 8 |
| **WT (cell-attached)** | Control | -29.8 ± 2.5 | 29.3 ± 2.5 | 9 | -25.9 ± 3.7 | 29.8 ± 3.7 | 7 |
|  | 0.1% DMSO | -34.2 ± 1.5 | 26.8 ± 1.5 | 4 | -25.6 ± 3.3 | 33.6 ± 3.5 | 5 |
|  | MLN4924 | -34.0 ± 1.3 | 25.6 ± 1.2 | 15 | -20.9 ± 3.2 | 33.1 ± 3.4 | 7 |
|  | Vector | -23.5 ± 1.8 | 26.5 ± 1.7 | 28 | -19.5 ± 3.2 | 28.5 ± 3.2 | 16 |
|  | Ub-K0 | -37.5 ± 2.6 | 28.6 ± 2.6 | 5 | -31.8 ± 3.4 | 30.1 ± 3.4 | 3 |
|  | DN-CUL4A | -29.1 ± 2.4 | 27.5 ± 2.4 | 20 | -22.4 ± 2.3 | 28.7 ± 2. 3 | 11 |
|  | DN-CUL4B | -28.8 ± 2.2 | 25.8 ± 2.1 | 16 | -25.3 ± 3.8 | 33.9 ± 4.1 | 11 |

***Supplementary Figure S1. Lack of effects of DN-CUL1/2/3/5 co-expression on CLC-1 protein level.*** (related to Figure 1)

Representative immunoblots showing the co-expression of Myc-CLC-1 with various Flag-DN-CUL constructs. Co-expression with the Flag vector was used as the control experiment. The gels were run under the same experimental conditions.


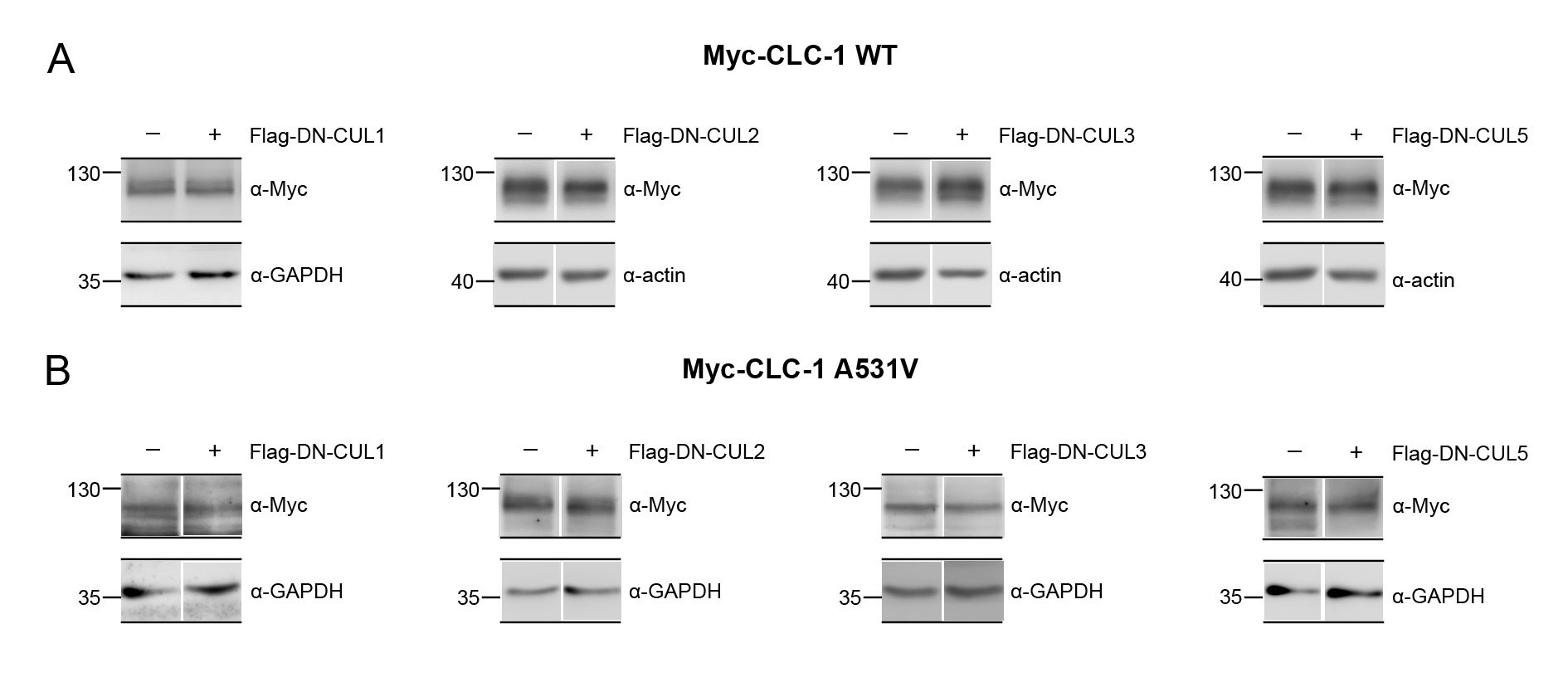


***Supplementary Figure S2. Uncropped images of the immunoblots presented in the main figures.*** (related to Figures 1-5)

(for Figure 1)


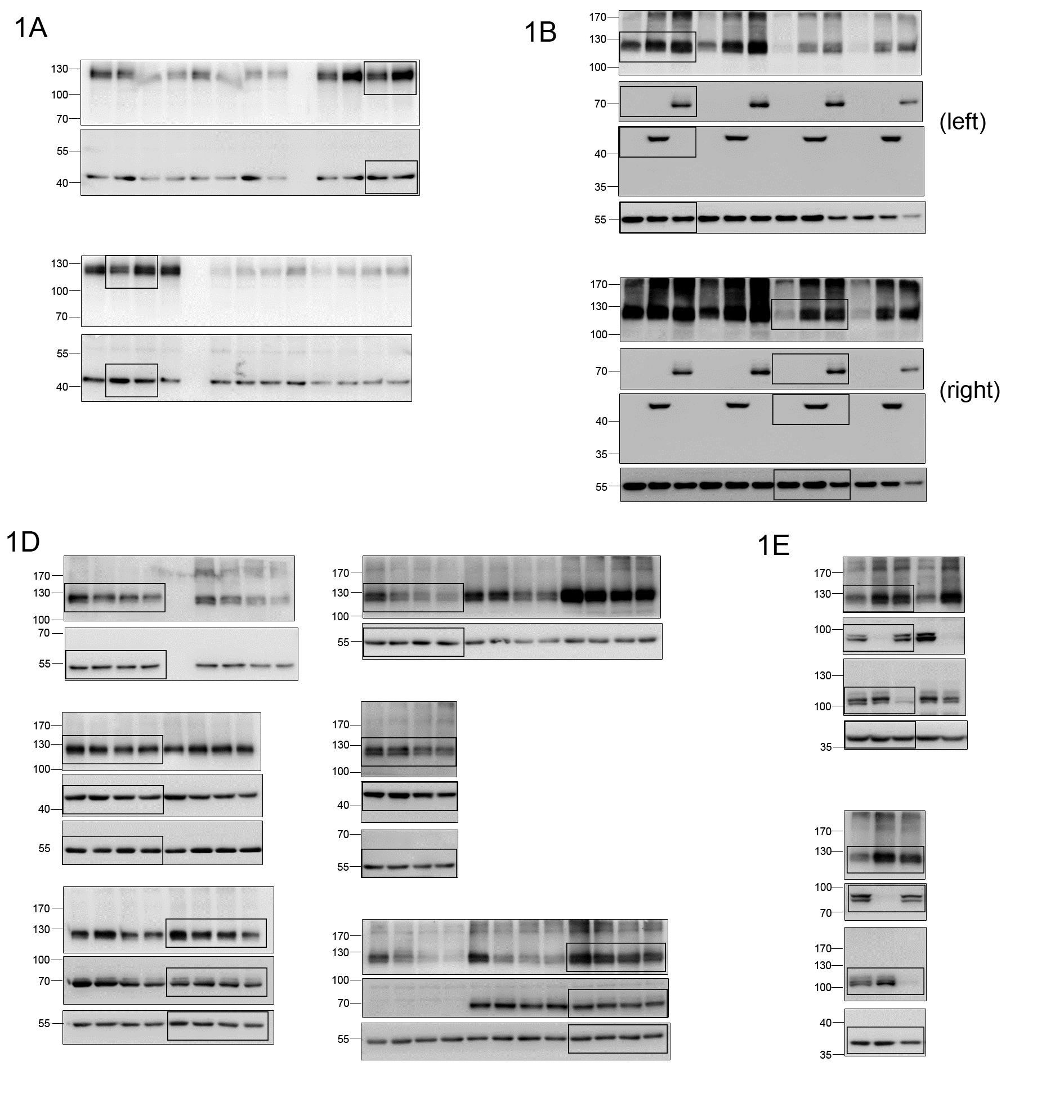


(for Figure 2)


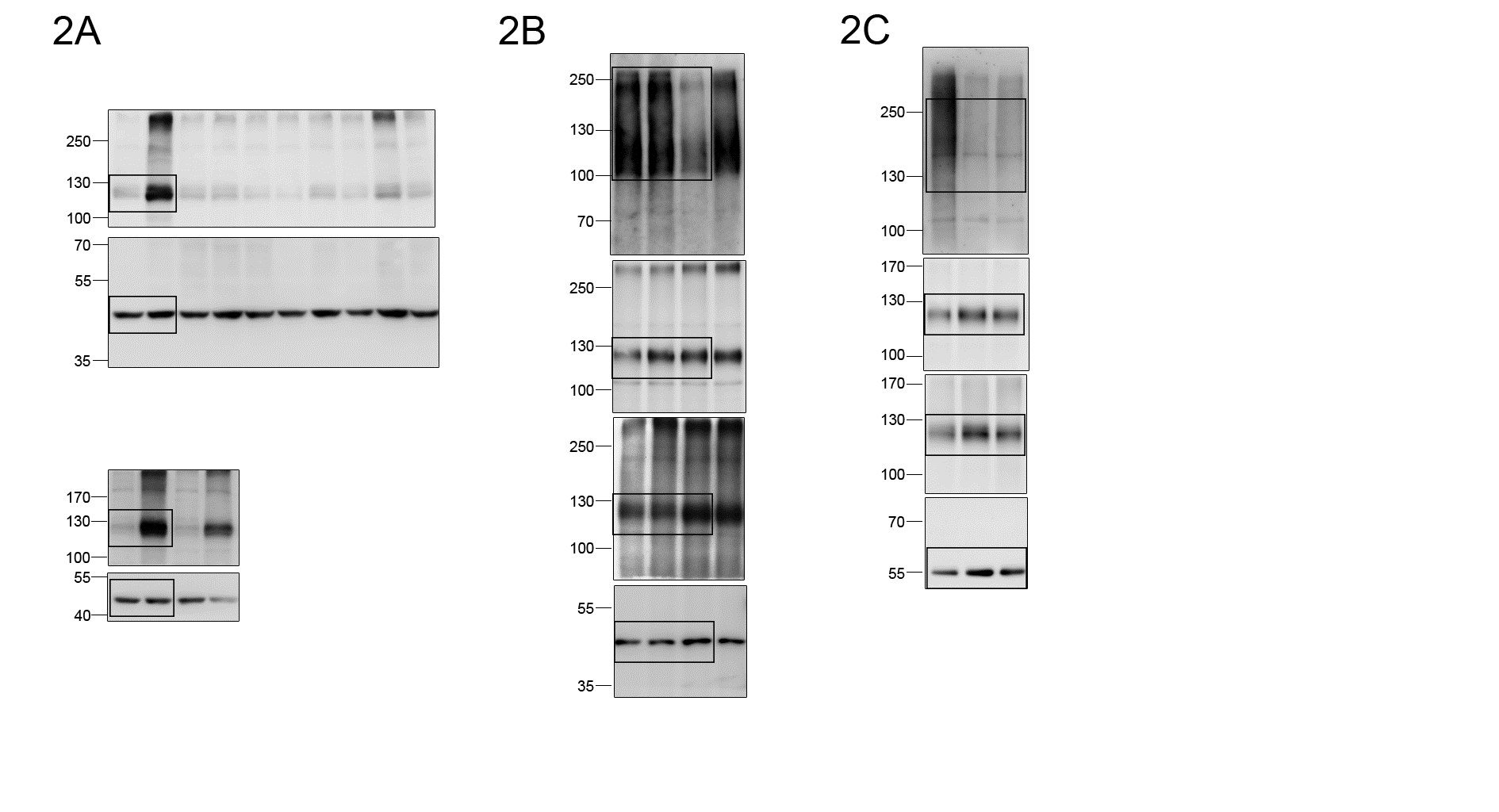


(for Figure 3)


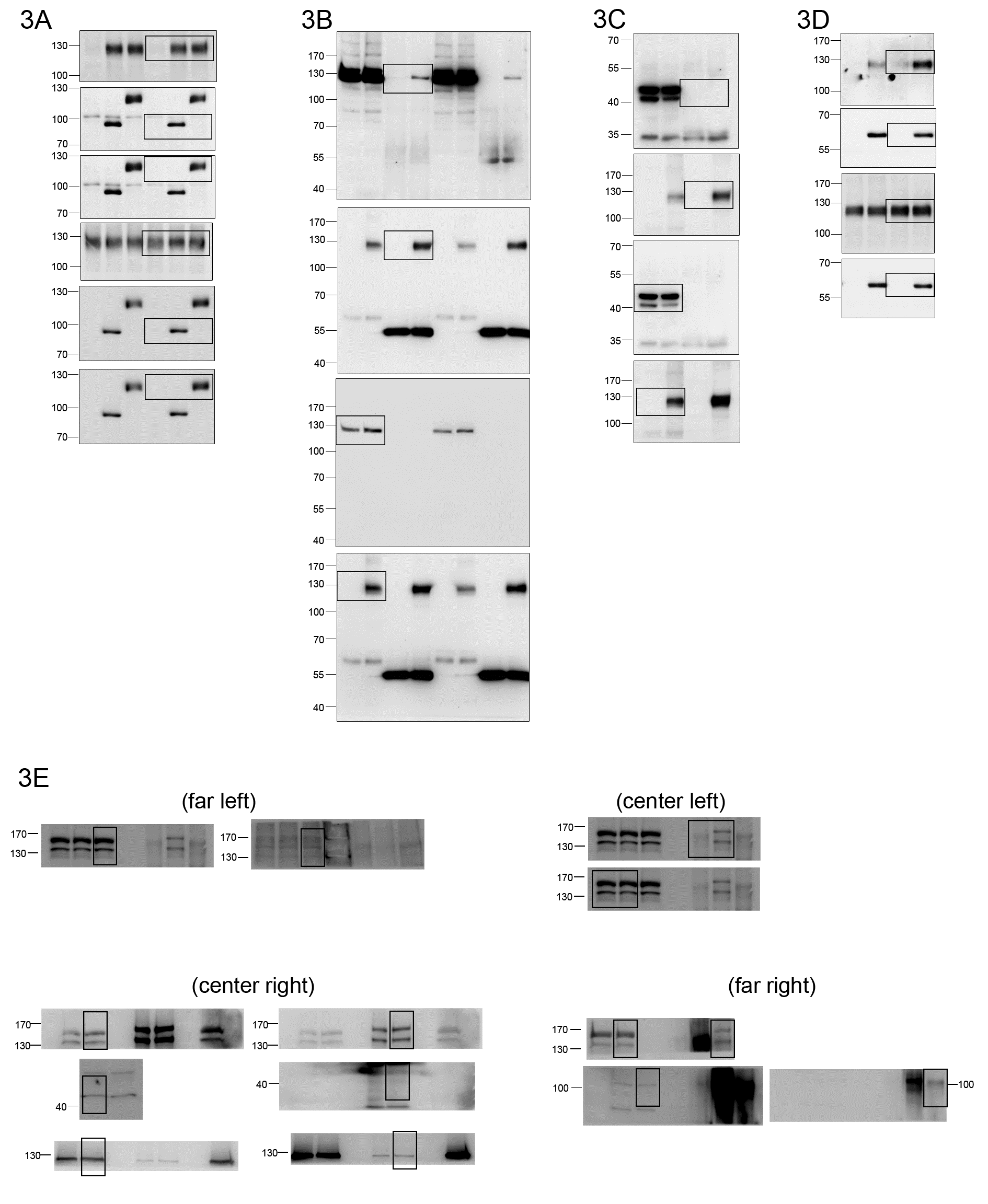


(for Figure 4)


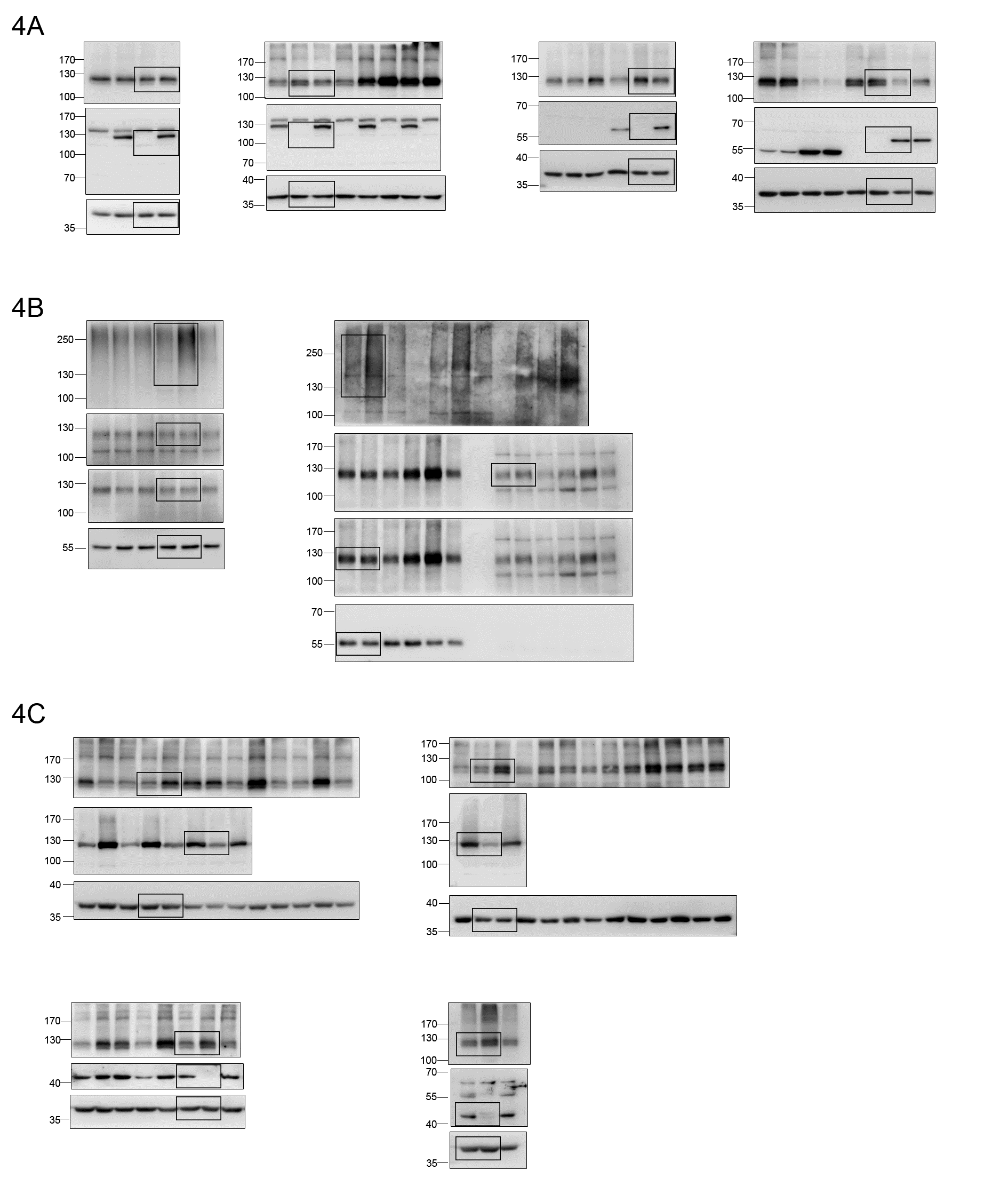


(for Figure 5)


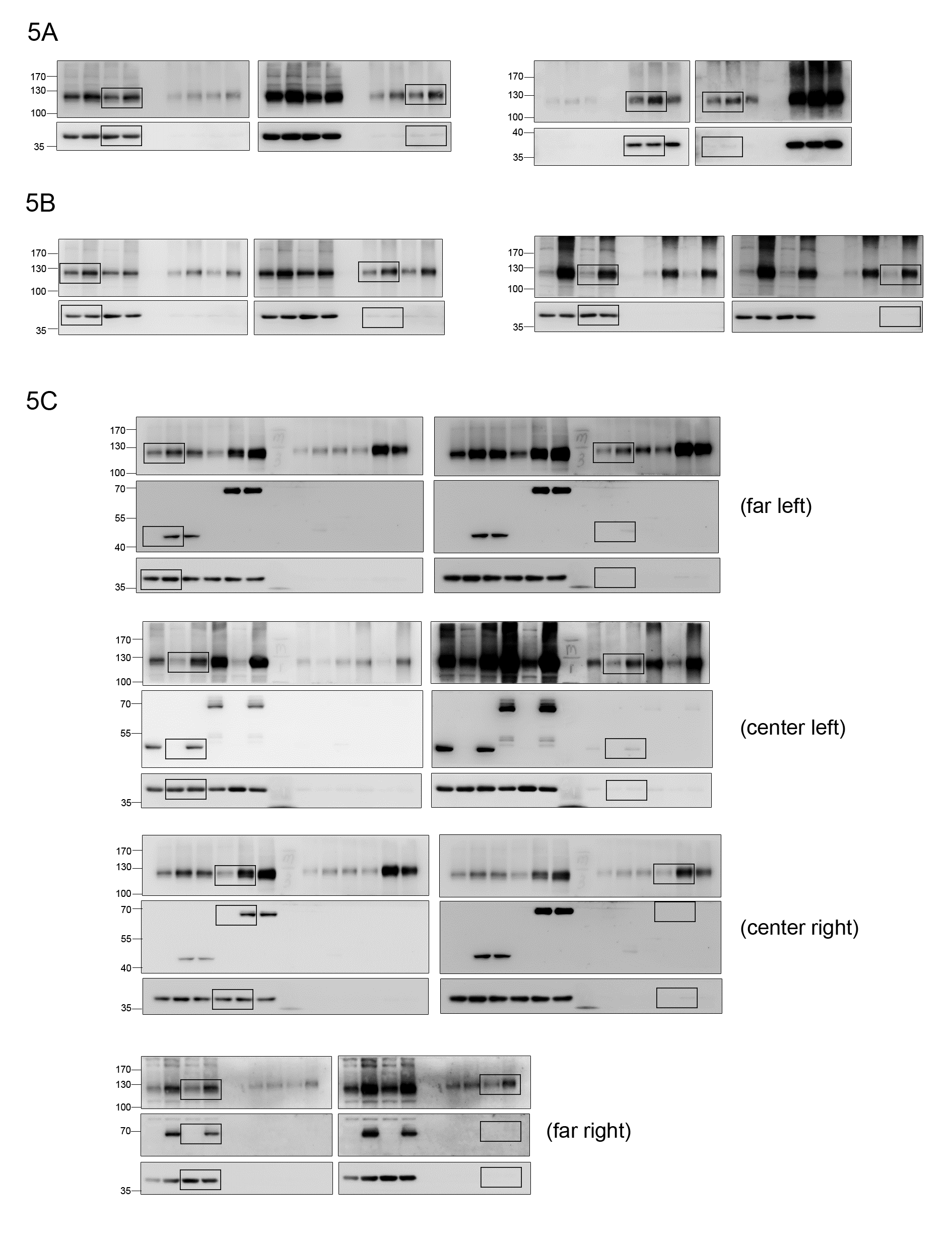


***Supplementary Figure S3. MLN4924 treatment of skeletal muscle explant enhances CLC-1 protein expression.*** (related to Figure 3)

Representative immunoblots showing the effect of MLN4924 treatment on CLC-1 protein level in skeletal muscle explant. The numbers denote the relative CLC-1 expression level with respect to the DMSO control. Dissected skeletal muscle (vastus lateralis) was adequately chopped and placed in DMEM in the 37ºC incubator for 1 hour. Tissues (100 mg) were then blotted dry on sterile filter paper and transferred to 24-well tissue culture plates for incubation in 1 ml DMEM (with 100 U/ml penicillin G and 100 mg/ml streptomycin) in the presence of 0 (DMSO), 0.5, 1, or 20 μM MLN4924 in the 37ºC incubator for 15 hours, followed by homogenization and immunoblotting analyses. The gels were run under the same experimental conditions.


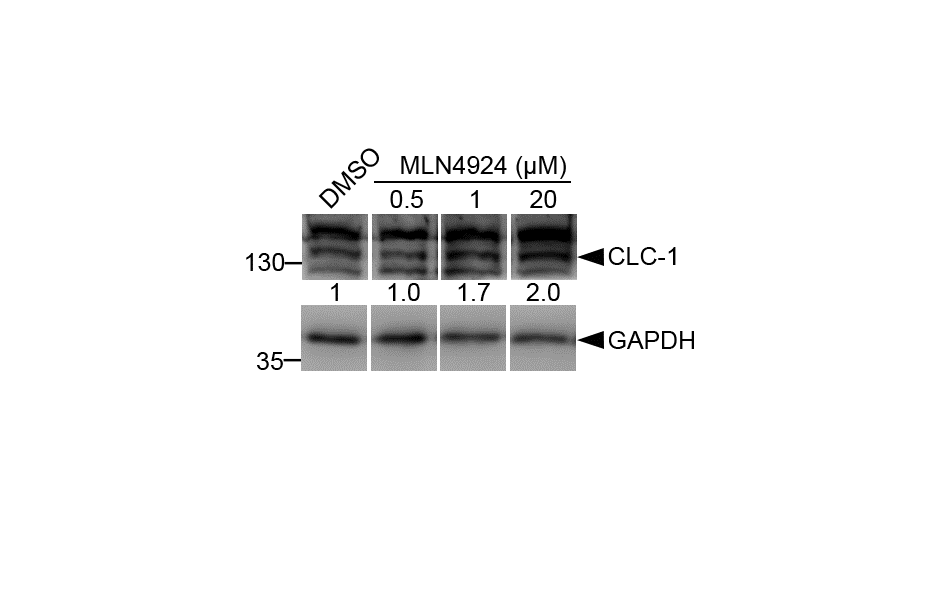


***Supplementary Figure S4. The protein half-life of CRBN is considerably shorter than that of CLC-1 channels.*** (related to Figure 4)

*(Left)* Representative immunoblots showing the protein turn-over time course of CLC-1 WT, A531V mutant, and CRBN in the presence of different treatment durations of 100 μg/ml cycloheximide (CHX). *(Right)* Protein densities were standardized with respect to the cognate actin signal, followed by normalization to the control at 0 hr. The estimated protein half-life for CRBN (n = 6) is about 0.6 hour. The gels were run under the same experimental conditions.


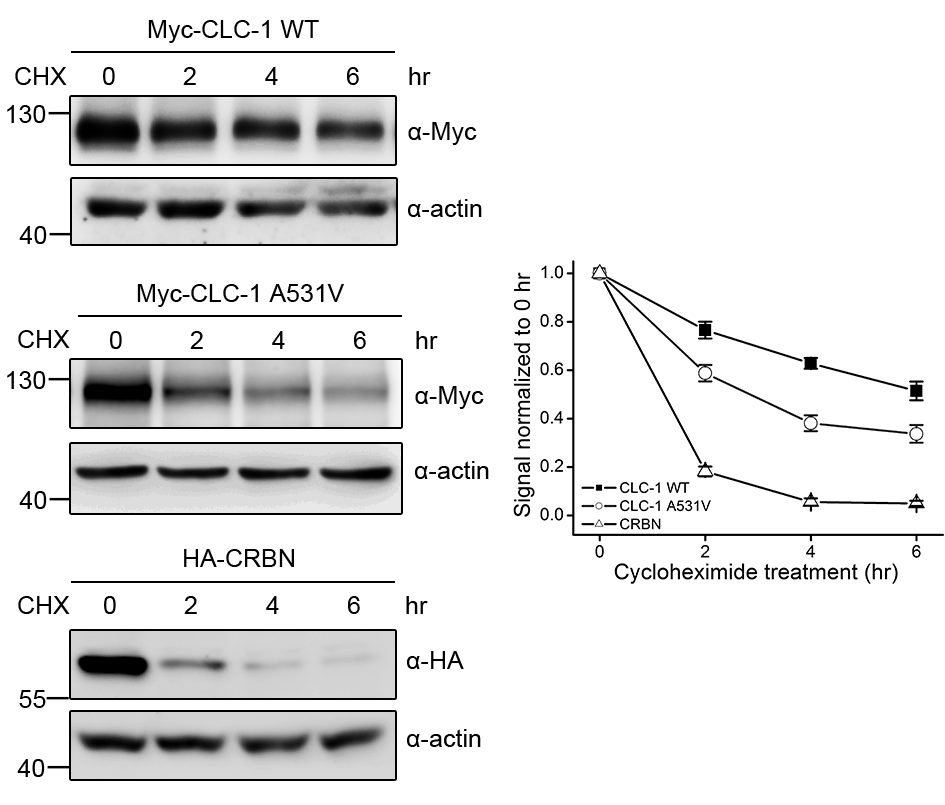


***Supplementary Figure S5. Further analyses of CLC-1 A531V whole-cell current recordings.*** (related to Figure 6)

***(A-B)*** Re-analyses of the CLC-1 A531V data in Figure 6: only those with peak instantaneous current amplitudes [at -140/-100 mV ***(A)*** or at -80/-60 mV ***(B)***] smaller than 10 nA were chosen for comparing whole-cell current densities. Asterisks denote a significant difference from the control condition (*, *t*-test: p < 0.05). ***(C)*** Representative current-voltage (I-V) curves from cells with peak current amplitudes (at -140 mV) close to/larger than 10 nA *(left)* or smaller than 10 nA *(right)*. Whole-cell recordings from a given cell were subject to the filtering frequencies 1, 2, 5, or 10 Hz (sampling rate = 2 x filtering frequency).


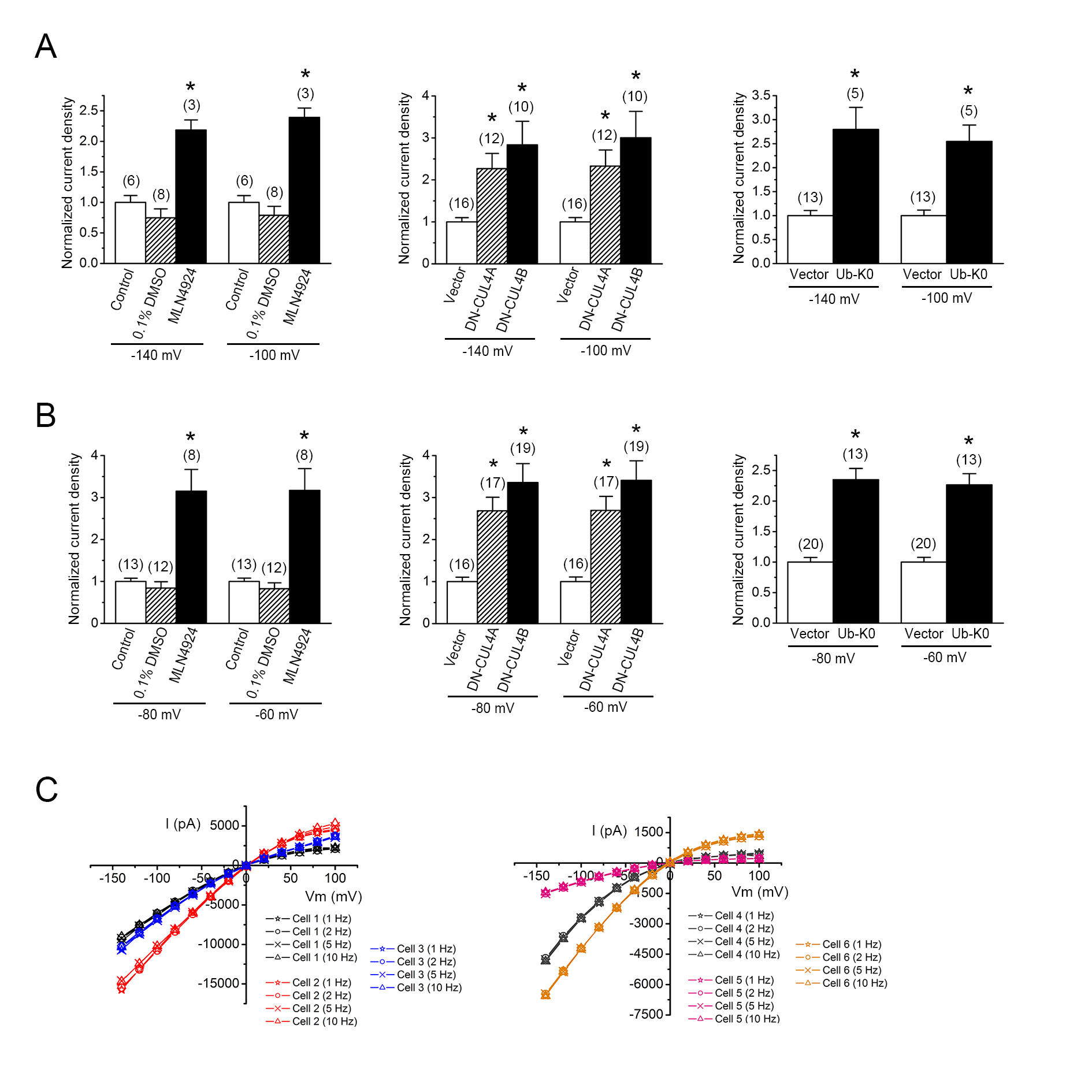


***Supplementary Figure S6. Suppression of CUL4A/B E3 ligase activity enhances the functional expression of CLC-1 WT channels.*** (related to Figure 6)

Electrophysiological analyses of Flag-CLC-1 WT channels in HEK293T cells. Treatment with 10 μM MLN4924 ***(A)*** or co-expression with Ub-K0 or DN-CUL4A/B ***(B)*** increased the current amplitude of the WT. *(Left)* Representative patch clamp recordings. Cell-attached patch clamp technique was employed since the functional expression of WT channels is much more robust than its mutant counterpart. *(Upper right)* Normalized instantaneous current amplitudes. *(Lower right)* Steady-state voltage-dependence properties. Asterisks denote a significant difference from the control condition (*, *t*-test: p < 0.05).


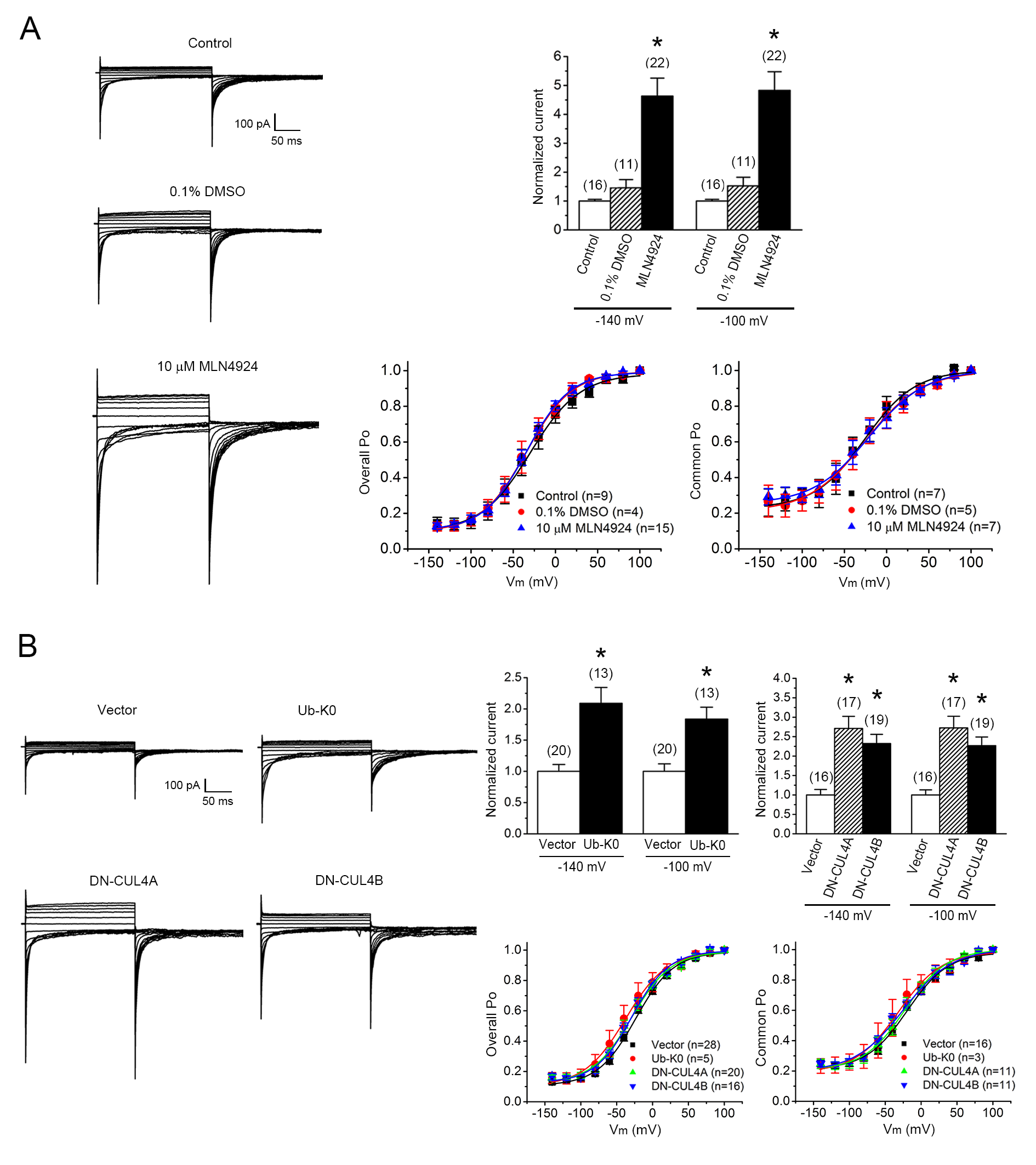

Supplement: Supporting Information [file srep10667-s1.doc]
